# Retraction: Fabrication of novel quantum dots for the estimation of COVID-19 antiviral drug using green chemistry: application to real human plasma

**DOI:** 10.1039/d4ra90133a

**Published:** 2024-11-08

**Authors:** Baher I. Salman, Adel Ehab Ibrahim, Sami El Deeb, Roshdy E. Saraya

**Affiliations:** a Pharmaceutical Analytical Chemistry Department, Faculty of Pharmacy, Al-Azhar University Assiut Branch Assiut Egypt bahersalman@azhar.edu.eg bahersalman2013@yahoo.com +201099031345; b Natural and Medical Sciences Research Center, University of Nizwa Birket Almawz P. O. box 33 Oman; c Department of Pharmaceutical Analytical Chemistry, Faculty of Pharmacy, Port Said University Port Said 42511 Egypt; d Institute of Medicinal and Pharmaceutical Chemistry, Technische Universitaet Braunschweig 38106 12 Braunschweig Germany

## Abstract

Retraction of ‘Fabrication of novel quantum dots for the estimation of COVID-19 antiviral drug using green chemistry: application to real human plasma’ by Baher I. Salman *et al.*, *RSC Adv.*, 2022, **12**, 16624–16631, https://doi.org/10.1039/D2RA02241A.

The Royal Society of Chemistry, with the agreement of the authors, hereby wholly retracts this *RSC Advances* article due to concerns with the reliability of the data.

The left side TEM image of Fig. 1a shows inconsistencies in the background that the authors have not been able to satisfactorily explain.

The PXRD spectrum in Fig. 1d contains repeating patterns and there are anomalies in the *X*-axis.

The EDX spectrum in Fig. 2a contains repeating patterns.

An independent expert was consulted who was not satisfied with the explanation provided by the authors.

Given the significance of the concern about the validity of the data, the findings presented in this paper are no longer reliable. The authors have cooperated throughout the investigation and have endeavoured to be transparent regarding the errors in the data.

Signed: Baher I. Salman, Adel Ehab Ibrahim, Sami El Deeb, Roshdy E. Saraya

Date: 9^th^ October 2024

Retraction endorsed by Laura Fisher, Executive Editor, *RSC Advances*

